# Editorial: Analysing writing processes of people with language, mental, cognitive or physical disorders

**DOI:** 10.3389/fpsyg.2023.1342846

**Published:** 2023-12-15

**Authors:** Luuk Van Waes, Åsa Wengelin, Ingrid Henriksson

**Affiliations:** ^1^Department of Management, Faculty of Business Economics, University of Antwerp, Antwerp, Belgium; ^2^Department of Swedish, Multilingualism, Language Technology, Faculty of Humanities, Gothenburg University, Gothenburg, Sweden; ^3^Speech and Language Pathology Unit, Institute of Neuroscience and Physiology, Sahlgrenska Academy at Gothenburg University, Gothenburg, Sweden

**Keywords:** writing processes, language disorders, mental disorders, hearing disorders, keystroke logging

This Research Topic highlights the analysis of writing processes of people with language, mental, cognitive or physical disorders, providing a range of studies that partially close the current gap in this area. The first papers address the characteristics of the writing processes of participants with language disorders in general and, more specifically, also, with dyslexia. The other papers focus on the writing of deaf and hard-of-hearing people and also persons with Alzheimer or other neurological/psychological issues. All together, the articles cover the lifelong span of writing, including participants between 7 and 88 years old with and without certain language-related disorders. We present both papers that observed participants with developmental disorders and those with acquired disorders. Table 1 shows an annotated overview of all the contributions to this Research Topic and their main perspectives.

**Table 1:** Matrix showing the different characteristics of each of the studies.

	**Disorder**	**Focus**	**Participants**	**Writing mode**	**Observation**	**Analysis**	**Task**
Critten et al.	Developmental Language Disorder (DLL)	Spelling • Morphology • Pauses	7–10 years (*n* = 3^*^33)	Handwriting	Eye tracking (eye and pen)	ANOVA • Correlation • Regression	Dictation
Pascual et al.	Struggling writers	Pause (thresholds)	8–11 years (*n* = 67 + 16)	Handwriting	Neo Smartpens •Handspy	Multilevel modeling (CCLMM-Bayesian)	Copy task (alphabet task)
Kraft	Reading and writing difficulties	Revisions	10–13 years (*n* = 12 + 16)	Speech to text • Keyboarding	Keystroke logging (Inputlog & Scriptlog) • Screen recording (camtasia)	ANOVA • Man–Whitney	Expository text
Olujić Tomazin et al.	Dyslexia	Triple task reactivity • Pauses • revisions	Adults (*n* = 20 + 20)	Keyboarding	Keystroke logging (Inputlog)	Bayesian mixed ANOVA	Narrative tasks
Gärdenfors and Johansson	Deaf and hard of hearing	Fluency • Pauses • Revision	10–12 years (*n* = 14 + 10 + 12)	Keyboarding	Keystroke logging (Scriptlog & Inputlog)	Correlation • Regression	Narrative stasks
Meulemans et al.	Alzheimer (MCI)	Fluency • Time on task • Pauses	62–87 years (*n* = 15 + 15)	Keyboarding	Keystroke logging (Inputlog)	Mixed-effect models	Picture discription task

In the first paper, Critten et al. report a study in which they compared the (hand)writing process of young children with Development Language Disorder (DLD), both with a group of age-matched and a group of younger children. The children finished a dictation spelling task in English, with a particular focus on morphological issues. They used the Eye and Pen device to observe the children's writing. One of their main perspectives to compare participant groups in this study, is related to a pause analysis at the root/suffix boundaries. The focus on pauses is central to their analysis, which is the case in almost all papers in this Research Topic (and also in the related literature; see also Table 1). Not surprising, as pauses are supposed to provide an (indirect) process-level marker for cognitive load in general and especially also for writing difficulties.

However, we should always bare in mind that pauses are not always easy to interpret, as they are an indirect measure of cognitive effort. Moreover, most pause analyses use a certain threshold, and up till now there is no real evidence based account to define this pause threshold. Pascual et al. tried to address this latter issue and set up an experiment to compare the pausing behavior of typically-developing vs. struggling writers. Controlling for a wide set of letter features, they attribute these features to handwriting processes for both groups of participants. Their article closes by providing a set of recommendations to define the pause threshold in handwriting studies.

While the two previous papers focused on the pausing behavior of struggling writers in handwriting, Kraft analyzed the revision behavior of children with and without reading and writing difficulties. For her study, she utilized speech-to-text (STT) technology as a writing input, in addition to keyboarding. The children were observed using keystroke logging and screen capturing. The underlying hypothesis was that struggling writers would benefit from STT as they experience difficulties with decoding and spelling.

Olujić Tomazin et al.—and all the other papers following in this Research Topic—used keyboarding too. In their paper, they present a methodological approach to evaluate the 'triple task' used in experiments involving adults with and without dyslexia. The triple task technique was introduced in writing research to (complementary) measure and assess the writers' cognitive load while producing texts. In this methodological study, they evaluate the reactivity of this triple task technique through a within and between comparison of participants with and without dyslexia writing a narrative text. The main process focus in their analysis is the writers' pausing and revision behavior.

Gärdenfors and Johansson also focus on pauses and revisions in the writing process, i.c. also while writing a narrative text. However, their study focused on another group of writers, viz. deaf and hard of hearing. These pupils' pausing and revision behavior was compared with age-matched hearing children of deaf adults that mastered sign language and age-matched hearing peers that did not master sign language. Using keystroke logging, they also explore a set of fluency measures to compare the writing process of the three groups.

Finally, Meulemans et al. present a study analyzing senior participants with alzheimer (mild cognitively impairment—MCI) compared to the writing behavior of a gender and an aged-matched group of healthy writers. The participants were observed while performing a set of picture description tasks. Keystroke logging was used to measure overall writing process measures and pausing behavior. The results indicate that monitoring writing behavior of MCI-patients could be a promising (complimentary) practice in detecting and monitoring cognitive impairment due to alzheimer.

The articles presented demonstrate that writing tasks, observation techniques, and process characteristics—such as pausing and revision behavior—enable researchers to identify differences in the writing processes of individuals with language, mental, cognitive or physical disorders. Moreover, also in the diagnosis of certain disorders, writing process analyses can be used to complement the currently used instruments. The studies can also be used to refine support measures in dealing with disorders. For instance, in school policies, certain facilities are often provided to support children with dyslexia in schools. Up till now, these policies are often based on findings articulated in reading research. As writing has become only more important in recent years (Brandt, 2014), writing research can certainly complement these findings, creating a better and broader basis for these proposed policies.

## Author contributions

LV: Writing—original draft, Writing—review & editing. ÅW: Writing—review & editing. IH: Writing—review & editing.
